# The temporal distribution of new H7N9 avian influenza infections based on laboratory-confirmed cases in Mainland China, 2013–2017

**DOI:** 10.1038/s41598-018-22410-w

**Published:** 2018-03-06

**Authors:** Zuiyuan Guo, Dan Xiao, Dongli Li, Yayu Wang, Tiecheng Yan, Botao Dai, Xiuhong Wang

**Affiliations:** 1 Department of Disease Control, Center for Disease Control and Prevention of Shenyang Military Region, Shenyang, China; 2 China National Clinical Research Center for Neurological Diseases, Beijing TianTan Hospital, Beijing, China; 3 The Emergency Center for Animals Disease of Liaoning Province, Shenyang, China

## Abstract

In this study, estimates of the growth rate of new infections, based on the growth rate of new laboratory-confirmed cases, were used to provide a statistical basis for in-depth research into the epidemiological patterns of H7N9 epidemics. The incubation period, interval from onset to laboratory confirmation, and confirmation time for all laboratory-confirmed cases of H7N9 avian influenza in Mainland China, occurring between January 2013 and June 2017, were used as the statistical data. Stochastic processes theory and maximum likelihood were used to calculate the growth rate of new infections. Time-series analysis was then performed to assess correlations between the time series of new infections and new laboratory-confirmed cases. The rate of new infections showed significant seasonal fluctuation. Laboratory confirmation was delayed by a period of time longer than that of the infection (average delay, 13 days; standard deviation, 6.8 days). At the lags of −7.5 and −15 days, respectively, the time-series of new infections and new confirmed cases were significantly correlated; the cross correlation coefficients (CCFs) were 0.61 and 0.16, respectively. The temporal distribution characteristics of new infections and new laboratory-confirmed cases were similar and strongly correlated.

## Introduction

In March 2013, a new type of avian influenza virus, H7N9, was isolated for the first time in China^1^. In the spring of that year, an outbreak of H7N9 influenza occurred in 10 provinces in central and eastern China^2^. Until June 30, 2017, the H7N9 virus has caused 5 nationwide outbreaks: January 2013 to September 2013 (134 cases, 45 deaths), October 2013 to September 2014 (306 cases, 131 deaths), October 2014 to September 2015 (219 cases, 102 deaths), October 2015 to September 2016 (114 cases, 47 deaths), and October 2016 to June 2017 (1058 cases, 221 deaths)^3,4^. The average case fatality risk of H7N9 infection is approximately 40%^3^, which is higher than the case fatality risk of SARS (11%)^5^ and lower than that of H5N1 (70%)^2^. The fifth epidemic was the most severe; cases were diagnosed in 20 provinces and the total number of cases exceeded the sum of the previous 4 epidemics^3^. These 5 epidemics occurred mostly in China’s Yangtze and Pearl River Deltas^3^. The genetic characteristics of the viral strains were similar in all 5 epidemics^6,7^. The main sites of infection were live poultry markets^8,9^. The main route of transmission was human contact with infected poultry or virus-contaminated environments; no connections were demonstrated between patients^7^, and only limited, non-sustained human-to-human transmission within individual regions was observed^10,11^.

A typical confirmed case of H7N9 avian influenza, from infection to diagnosis, progresses through the following 3 stages: (1) From infection by exposure to the pathogen to symptom presentation, after an incubation period; (2) from symptom presentation to hospital admission and treatment; and (3) from hospitalization to laboratory confirmation by a state-certified network laboratory. The state-certified network laboratory reports each case, via the pneumonia of unknown etiology (PUE) surveillance system, to the Chinese Center for Disease Control and Prevention (China CDC)^12^. A study conducted by Cowling *et al*.^2^ showed that the mean incubation period of H7N9 is 3.1 days and follows a Weibull distribution, and that the median interval from onset to laboratory confirmation is 8.3 days and follows a log-normal distribution. These study results provide an important basis for our further investigations.

Because infection occurs before the diagnosis is made, assessing the epidemic trend using the growth rate of confirmed cases is imprecise; we consider the growth rate of new infections an important indicator of the seriousness of an epidemic. However, the exact time that a patient becomes infected is difficult to ascertain, mainly because patients themselves do not know exactly when they became infected; most can give only a rough estimate, and some are unclear even about this. Because the exact time that infection occurs cannot be obtained through epidemiological surveys, the use of mathematical models to arrive at a reasonable estimate is a good choice.

A number of researchers have used mathematical models to provide a quantitative description of the epidemiological characteristics of H7N9 epidemics^13–19^. For example, Zhang *et al*.^13^ established a dynamic model including migratory birds, resident birds, domestic poultry, and human populations; they concluded that migrant birds were most likely the original source of infection. Lin *et al*.^14^ modelled chicken-to-chicken transmission and found that environmental transmission via viral shedding by infected chickens contributed to the spread of the virus. These studies help us to understand the prevalence of the virus among poultry and the mechanisms of its transmission from poultry to humans. According to the transmission route of H7N9 avian influenza, each infected individual is relatively independent, the dynamic model of infectious disease spread cannot be used. Quantitative analysis of epidemic spread within human communities is crucial for a deeper understanding of the mechanisms by which epidemics spread. However, no studies have yet been published on the evolutionary mechanism of the entire process from infection to symptom onset to diagnosis.

The present study used statistical data on the temporal distribution of laboratory-confirmed cases and applied the theory of stochastic processes to reveal the transmission mechanisms of this epidemic among human communities. In the study, we performed a quantitative analysis of the temporal distribution pattern for the expected values and 95% confidence intervals (CIs) of the growth rate of new infections. Therefore, this study has important implications for a deeper understanding of the onset and progression of H7N9 avian influenza epidemics and for a more precise description of its temporal distribution patterns. Its results can be used for the timely assessment of the effects of a series of government-instituted interventions and for exploring causal relationships between the time series of new infections and new confirmed cases, and provide a statistical basis for in-depth analysis of the impact of human and natural factors on the epidemic.

## Methods

### Data sources

The confirmation dates of all new H7N9 confirmed cases between January 2013 and January 2017 in Mainland China were obtained from the China CDC; data between February and June 2017 were obtained from the EMPES-i georeferenced disease data repository compiled by the Food and Agricultural Organization^4^. All cases were confirmed as H7N9 by local and/or provincial influenza network laboratories. H7N9 avian influenza is a category B infectious disease according to China’s notifiable infectious diseases classification; hence, cases confirmed by network laboratories need to be reported immediately using the PUE surveillance system^12^.

### Temporal distribution of the number of new infections

Our goal was to treat all laboratory-confirmed cases between January 2013 and June 2017 as a sample with number *N*, and to use this sample to estimate the temporal distribution for the growth rate of new infections.

First, we used Bayes theorem to analyze the probability of infection and subsequent diagnosis given that each confirmed case experiences 2 stages between infection and diagnosis: The first is between infection and onset (the incubation period), the second is between onset and laboratory confirmation. The incubation period follows a Weibull distribution^2^, with a probability density function expressed as *f*(∙). The onset-to-laboratory confirmation interval follows a log-normal distribution^2^, with a probability density function expressed as *g*(∙). These 2 time periods are mutually independent. The conditional probability, $\delta (T|{t}_{i})$, indicating the probability of an infected individual receiving laboratory-confirmation after a duration of *T* under the condition that infection occurred at time point *t*_*i*_, was expressed as convolution equation (1),

1 $$\delta (T|{t}_{i})={\int }_{0}^{T}f(s)g(T-s){\rm{d}}s\,\,0 < s < T$$

where $s$ denotes the duration of the incubation period and $T-s$ indicates the onset-to-laboratory-confirmation duration. As laboratory confirmation could happen at any time of the day, *T* includes a range of values, $[{T}_{0},{T}_{1}]$, where *T*_0_ and *T*_1_ denote the beginning and the end of the day, respectively. The conditional probability $\tilde{\delta }(T|{t}_{i})$ was used to indicate the probability of an infected individual having received laboratory-confirmation during the period ${T}_{0}$~${T}_{1}$ given that infection occurred at time point ${t}_{i}$ (equation 2),

2 $$\tilde{\delta }(T|{t}_{i})={\int }_{{T}_{0}}^{{T}_{1}}\delta (T|{t}_{i})dT$$

where $p({t}_{i})$ denotes the probability that a susceptible person is infected at time *t*_*i*_ and $P({t}_{i},{T}_{i})$ denotes the probability of receiving laboratory confirmation during day *T*_*i*_ given that a susceptible person is infected at time *t*_*i*_ and after a period of ${T}_{i}$. Using Bayes theorem, $P({t}_{i},{T}_{i})$ was expressed as equation (3),

3 $$P({t}_{i},{T}_{i})=p({t}_{i})\tilde{\delta }({T}_{i}|{t}_{i})$$

Thus, we could construct a likelihood function $L(\cdot )$:

4 $$\begin{array}{ccc}L({X}_{1},\cdots ,{X}_{N}) & = & \prod _{i=1}^{N}P({t}_{i},{T}_{i})\\  & = & \prod _{i=1}^{N}p({t}_{i})\tilde{\delta }({T}_{i}|{t}_{i})\\  & = & \prod _{i=1}^{N}p({t}_{i})\prod _{i=1}^{N}\tilde{\delta }({T}_{i}|{t}_{i})\end{array}$$

Where ${X}_{i}$ ($i=1,\cdots ,N$) denotes the *i* ^th^ infection that occurs. We then calculated the growth rate of new infections by analyzing $\prod _{i=1}^{N}p({t}_{i})$ using stochastic process theory.

**Figure 1:**
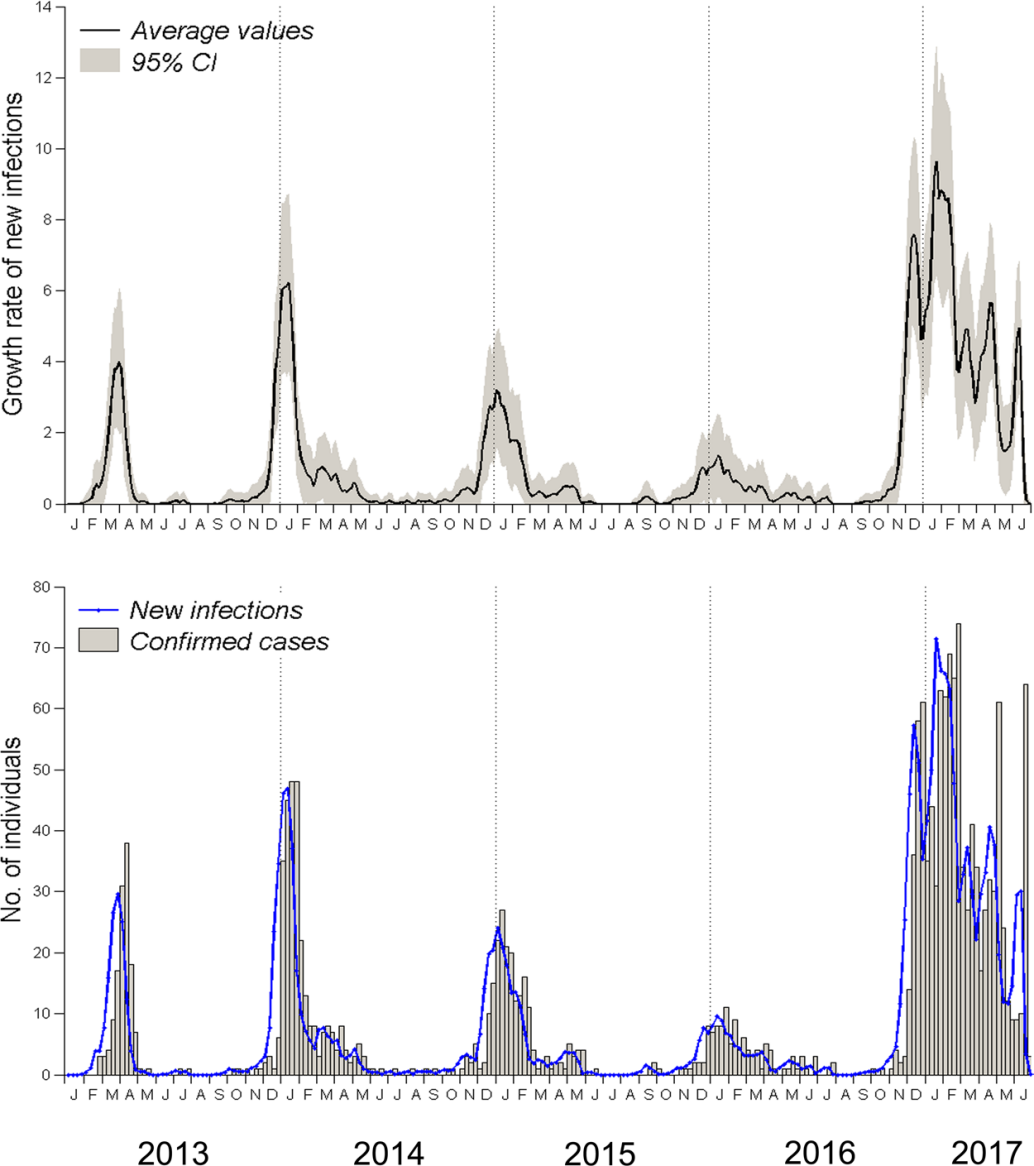
Temporal distribution of new infections and new confirmed cases of avian influenza A (H7N9) virus infection. (**A**) Temporal distribution of the growth rates of new infections. The black solid line represents the mean values, the grey area represents 95% confidence intervals. (**B**) Comparison of the number of new infections and new confirmed cases. The histograms show the number of new reported confirmed cases; the blue line represents the expected number of new infections.

The main transmission route of H7N9 is human contact with infected poultry or virus-contaminated environments; the virus is not continually spread among people, rather, susceptible individuals are randomly infected. The frequency of contact is variable at different times of the year, so the growth rate of new infections is also variable, being lower in summer than that in winter and spring (Fig. 1). We assumed that continuous emergence of infections represents a series of independent random events.

$\{N(\tau ),\tau \ge 0\}$ denotes the process of counting H7N9 infections within a short time period $\tau$. Based on the analysis above, $N(\tau )$ can be regarded as a Poisson process with rate *λ*, and the time interval of any 2 consecutive infections will follow independent exponential distribution^20^. For a time interval of length $\tau$, the probability $P({X}_{1},\cdots ,{X}_{m};\lambda ,\tau )$ that *m* infections will occur consecutively at time points ${s}_{1}$, …, ${s}_{m}$ can be expressed as:

5 $$\begin{array}{rcl}P({X}_{1},\ldots ,{X}_{m};\lambda ,\tau ) & = & P\{{\rm{m}}\,{\rm{infections}}\,{\rm{in}}\,[0,{s}_{m}]\}P\{{\rm{none}}\,{\rm{in}}\,({s}_{m},\tau ]\}\\  & = & \lambda {e}^{-\lambda {s}_{1}}\lambda {e}^{-\lambda ({s}_{2}-{s}_{1})}\cdots \lambda {e}^{-\lambda ({s}_{m}-{s}_{m-1})}{e}^{-\lambda (\tau -{s}_{m})}\\  & = & {\lambda }^{m}{e}^{-\lambda \tau }\end{array}$$

Because the growth rate of new infections varied during the period 2013~2017, and for reasons of convenience in the calculations, we assumed a constant growth rate over a short period of time. Hence, the rate changed over the course of a few consecutive time intervals of moderate length (if the interval is too long the result will be inaccurate, and if it is too short it will increase the amount of calculation required). We used intervals of an eighth of a month. There were 54 months between January 2013 and June 2017, giving 432 intervals. The growth rate of new infections in each interval was expressed as ${\lambda }_{k}$ $(k=1,\cdots ,432)$, and the length was a constant value, $\tau$. The entire process of an infection appearing can be viewed as a continuous-time Markov chain with different rates and as a pure birth process^20^ with states of ${X}_{0}$, ${X}_{1}$, …, ${X}_{N}$. Hence, $p({t}_{i})$ can be converted to the probability of when the system transitions from state ${X}_{i-1}$ to state ${X}_{i}$ at time ${t}_{i}$, as follows:

6 $$p({t}_{i})=p\{X({t}_{i})=i|X({t}_{i-1}\le t < {t}_{i})=i-1\}$$

7 $$p({t}_{i})=\{\begin{array}{cc}{\lambda }_{k}{e}^{-{\lambda }_{k}({t}_{i}-{t}_{i-1})} & {\lambda }_{k}:[{t}_{i-1},{t}_{i}]\\ {e}^{-{\lambda }_{k-1}(t^{\prime} -{t}_{i-1})}{\lambda }_{k}{e}^{-{\lambda }_{k}({t}_{i}-t^{\prime} )} & \begin{array}{c}{\lambda }_{k-1}:[{t}_{i-1},t^{\prime} ),{\lambda }_{k}:[t^{\prime} ,{t}_{i}]\end{array}\end{array}$$

Based on the above analysis, $\prod _{i=1}^{N}p({t}_{i})$ is associated with ${\lambda }_{k}$ (equation 8).

8 $$\begin{array}{rcl}\prod _{i=1}^{N}p({t}_{i}) & = & \prod _{k=1}^{432}P({X}_{1},\ldots ,{X}_{{m}_{k}};{\lambda }_{k},\tau )(\sum _{k=1}^{432}{m}_{k}=N)\\  & = & \prod _{k=1}^{432}{\lambda }_{k}^{{m}_{k}}{e}^{-{\lambda }_{k}\tau }\end{array}$$

We calculated the value of ${\lambda }_{k}$ using the maximum likelihood; equation (9) was derived by incorporating equation (8) into equation (4).

9 $$\begin{array}{rcl}L(\widehat{\lambda },\tau ) & = & \mathop{\max }\limits_{\lambda ,\tau  > 0}L(\lambda ,\tau )\\  & = & \mathop{\max }\limits_{\lambda ,\tau  > 0}\prod _{i=1}^{N}p({t}_{i})\prod _{i=1}^{N}\tilde{\delta }({T}_{i}|{t}_{i})\\  & = & \mathop{\max }\limits_{\lambda ,\tau  > 0}\prod _{k=1}^{432}{\lambda }_{k}^{{m}_{k}}{e}^{-{\lambda }_{k}\tau }\prod _{i=1}^{N}\tilde{\delta }({T}_{i}|{t}_{i})\end{array}$$

After logarithmic transformation of equation (9), a very concise result could be obtained by taking the partial derivative of ${\lambda }_{k}$:

10 $${\lambda }_{k}=\frac{{m}_{k}}{\tau }$$

Where ${\lambda }_{k}$ is the growth rate of new infections.

On the basis of the above analysis, we calculated expected values and 95% CIs for ${\lambda }_{k}$ as follows: For each confirmed case, according to a uniform distribution, we randomly selected a time point on the day of laboratory confirmation as the confirmation time. Then, the Markov chain Monte Carlo method was applied to generate random numbers that fitted the probability density function *δ*(∙)^21^; these numbers represent the lengths of time from infection to laboratory confirmation. As the time of laboratory confirmation is known, the estimated time of infection can be calculated. After computing the infection times of all confirmed cases, counting was performed on 432 time intervals with length $\tau$ to sequentially obtain ${m}_{k}$, and hence to work out the growth rate of new infections ${\lambda }_{k}$ of one calculation. The bootstrap method was used to repeat this step 1000 times to obtain the mean values and 95% CIs for ${\lambda }_{k}$. The above method was implemented using MATLAB and Statistics Toolbox Release 2012a (The MathWorks, Inc., Natick, Massachusetts, USA).

### Correlation test of time series for new infections and new confirmed cases

Based on the above calculations, the expected number of new infections in quarters of a month can be obtained. These numerical values constitute the time series $$X=\{X(t),\begin{array}{c}t\ge 0\end{array}\}$$, and new confirmed cases will constitute the time series $$Y=\{Y(t),\begin{array}{c}t\ge 0\end{array}\}$$. $X$ was taken as the covariant variable and $Y$ as the dependent variable. The aim was to test the CCF at a lag of *k*, ${r}_{k}(X,Y)$, to verify the correlation between the 2 and to analyze whether the former was causing fluctuations in the latter.

11 $${r}_{k}(X,Y)=\frac{\sum ({X}_{t}-\overline{X})({Y}_{t-k}-\overline{Y})}{\sqrt{\sum {({X}_{t}-\overline{X})}^{2}}\sqrt{\sum {({Y}_{t}-\overline{Y})}^{2}}}\,\,k=1,2,\cdots $$

As these 2 time-series have strong autocorrelation (Fig. 1), if the correlation of the 2 processes is evaluated directly by calculating the CCFs, a false conclusion may be obtained (spurious correlation)^22^. Hence, the autocorrelation should be extracted from their respective series—pre-whitening is a precise tool for achieving this aim^22^. As the 2 series showed significant seasonal fluctuations, seasonal differencing was performed. After differencing, the series remained non-stationary; hence, first order differencing was performed to obtain 2 stationary time-series with a mean value of 0. Then, the same filter, based on the first series (an autoregressive model), was applied to pre-whiten the 2 time-series, followed by calculating the CCFs of the pre-whitened series^22^. The above method was implemented using R, version 3.1.0 (http://www.R-project.org/).

## Results

### Temporal distribution of new infections

Given that the incubation period of H7N9 fits a Weibull distribution (mean 3.1 days, standard deviation 1.4 days)^2^, we could work out the shape (2.4) and scale (3.5) parameters. As the onset-to-laboratory-confirmation interval fits a log normal distribution (median 8.3 days, 95% CI 7.3–9.5 days)^2^, we could work out the mean (2.1 days), standard deviation (0.6). Calculations were performed according to the methods described by JD Chen^23^.

The temporal distributions of the expected number of new infections and new confirmed cases showed a similar patterns of variation. Both displayed significant seasonal fluctuations and autocorrelation. Figure 1 shows that the first cases of infection appeared in Mainland China in February 2013. The number of infections increased drastically, reached a peak in March 2013, and then decreased drastically. From May to September 2013, infections were sporadic. The number of infections gradually increased from October through November 2013, then markedly increased in December, reaching a peak in January 2014, thereafter drastically decreasing. The epidemic stabilized from March through September 2014. Thereafter, the pattern of temporal distribution was essentially similar to what was observed in 2014, i.e. a high incidence in winter and spring with a low incidence in summer and autumn. The fifth epidemic was the most serious, with the number of infections increasing drastically in October 2016 and reaching a peak in January 2017; an estimated 229 new infections occurred in January 2017. Thereafter, the number of cases gradually decreased, but still fluctuated at a high level. From 2013 through May 2017, the expected numbers of infections annually were 212, 305, 171, 324, and 755. The average numbers of new infections per month are presented in Table 1.

**Table 1:** The average numbers of new infections per month during the period 2013~2017.

Years	Jan	Feb	Mar	Apr	May	Jun
2013	0	9	80	42	1	1
2014	147	27	27	15	8	1
2015	76	34	8	10	9	1
2016	33	19	13	5	8	4
2017	229	205	122	141	58	63
**Years**	**Jul**	**Aug**	**Sep**	**Oct**	**Nov**	**Dec**
2013	2	0	1	3	5	69
2014	1	2	2	4	11	61
2015	0	0	4	1	5	23
2016	3	0	1	4	45	190

### Correlation test of time series for new infections and new confirmed cases

**Figure 2:**
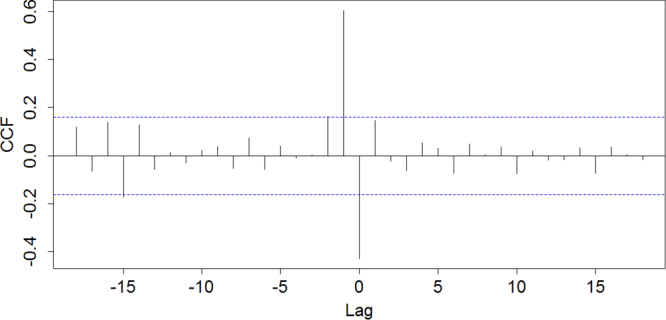
The cross-correlation graph for differenced and pre-whitened times series of new infections and new confirmed cases. The black vertical lines represent the cross-correlation coefficients; the two blue dotted lines represent 95% confidence interval.

Seasonal and first order differencing were performed on the time series of new infections and new confirmed cases, respectively. Then, pre-whitening (a nineteenth-order-autoregressive model was selected according to the Akaike information criteria) was performed, followed by computation of the CCFs. Figure 2 shows that 2 positive CCFs were obtained: When the lags were −2 and −1, the CCFs were 0.16 and 0.61, respectively. When the lag was 0, the CCF obtained was −0.43. In addition, when the lag was −15, there was a marginally significant negative CCF, which we believe to be a false alarm. This is because when calculating the CCFs for a total of 37 samples, we expect there to be an average of 37 × 0.05 = 1.85 false alarms. Based on the mean value of 1 interval (7.5 days), the mean duration from infection to laboratory confirmation, and the standard deviation, the 2 significant positive CCFs are at −2 and −1 time units.

## Discussion

Since the outbreak of the H7N9 avian influenza epidemic in multiple provinces across China in March 2013, researchers began conducting scientific research from different epidemiological perspectives. For example, Yuan *et al*.^24^ conducted sampling in live-poultry markets; detection of viral RNA demonstrated that live-poultry markets were the main sites at which H7N9 infection. Lin *et al*.^13^ and Zhang *et al*.^14^ established mathematical models to explore viral transmission mechanisms within different populations. Cowling *et al*.^2^, Wang *et al*.^3^, and Bui *et al*.^18^ provided detailed descriptions on the temporal, spatial, and population distributions of the epidemics. These studies laid the foundation for investigating the epidemiological characteristics of the H7N9 epidemics. Compared with these published studies, the innovative points lie in our analysis of the transmission mechanisms in the human population from a stochastic perspective, according to the transmission characteristics of H7N9. The occurrence of infection was treated as a random event produced by a Poisson process, and the rate of infection changed with time. Therefore, the entire epidemic process can be regarded as the connection of multiple Poisson processes occurring at different rates. We established a relationship between the probability of infection and a continuous time Markov chain; the growth rate of new infections was then obtained.

The initial stage of an infectious disease is effective contact with the pathogen. The growth rate of new infections reflects the frequency at which susceptible individuals come into contact with the virus: The more frequent the contact, the faster the rate of new infections increases. Therefore, we believe that this rate is the earliest and most accurate reflection of the severity of the epidemic. Although changes in the number of confirmed cases can reflect the temporal distribution features of the epidemic, laboratory confirmation of the diagnosis is delayed by a period of time greater than that of infection; in our study, the average delay was 13 days (standard deviation, 6.8 days). Therefore, evaluating the epidemic’s development trend using the temporal distribution of confirmed cases is imprecise. This study aimed to analyze the epidemic trend more accurately and to establish a more precise statistical foundation for analyzing the impact of various prevention and control measures adopted by China, and meteorological and environmental factors, on the epidemic. For example, some influencing factors—such as closing and regular disinfection of live poultry markets, atmospheric temperature, rainfall, and the number of migratory birds—can be evaluated more timely and accurately by judging the growth rate of new infections.

From Fig. 1B, we intuitively observe that there is a relatively strong correlation between the time series of infections and confirmed cases. However, this might not be a true correlation; a spurious correlation may exist. By calculating the CCFs after pre-whitening, we found significant positive correlation between the 2 time series, with the lags of −1 and −2 time units. Based on this, we can conclude that the occurrence of infections will lead to the occurrence of confirmed cases. Furthermore, the results verified that the establishment of random models is consistent with objective facts. With a lag of 0, a significant negative CCF is obtained (Fig. 2); this does not imply a true negative correlation between the 2 time series. Figure 1B shows that the time series of infections was smoother than that of confirmed cases, with opposite trends of change at certain time points.

Predicting epidemic trends through a mathematical model can help us to adopt more effective preventive measures to control potential epidemics. However, our model can only estimate the growth rate of infections in the past based on existing confirmed cases, i.e., it can only perform a retrospective analysis of epidemics; it is not predictive. More in-depth research on the transmission mechanisms of epidemics is required to establish new mathematical models that may require the inclusion of diverse factors such as immunization, closure of live-poultry markets, regular environmental disinfection, and other human interventions.
